# ALOX5AP is a new prognostic indicator in acute myeloid leukemia

**DOI:** 10.1007/s12672-023-00826-9

**Published:** 2023-11-23

**Authors:** Xin-Yi Chen, Xiang-Mei Wen, Wei Zhao, Ming-Qiang Chu, Yu Gu, Hai-Hui Huang, Qian Yuan, Zi-Jun Xu, Jun Qian, Jiang Lin

**Affiliations:** 1 Laboratory Center, Affiliated People’s Hospital of Jiangsu University, Zhenjiang, Jiangsu China; 2 Zhenjiang Clinical Research Center of Hematology, Zhenjiang, Jiangsu China; 3 Department of Hematology, Affiliated People’s Hospital of Jiangsu University, Zhenjiang, Jiangsu China

**Keywords:** ALOX5AP, Acute myeloid leukemia, DNA methylation, Gene expression, Prognosis

## Abstract

**Background:**

The overexpression of ALOX5AP has been observed in many types of cancer and has been identified as an oncogene. However, its role in acute myeloid leukemia (AML) has not been extensively studied. This study aimed to identify the expression and methylation patterns of ALOX5AP in bone marrow (BM) samples of AML patients, and further explore its clinical significance.

**Methods:**

Eighty-two de novo AML patients and 20 healthy donors were included in the study. Meanwhile, seven public datasets from Gene Expression Omnibus (GEO) and The Cancer Genome Atlas (TCGA) were included to confirm the alteration of ALOX5AP. Receiver operating characteristic (ROC) curve analysis was applied to determine the discriminative capacity of ALOX5AP expression to discriminate AML. The prognostic value of ALOX5AP was identified by the Kaplan–Meier method and log-rank test. It was further validated in four independent cohorts (n = 1186). Significantly different genes associated with ALOX5AP expression were subsequently compared by LinkedOmics, and Metascape database.

**Results:**

The level of ALOX5AP expression was significantly increased in bone marrow cells of AML patients compared with healthy donors (*P* < 0.05). ROC curve analysis suggested that ALOX5AP expression might be a potential biomarker to discriminate AML from controls. ALOX5AP overexpression was associated with decreased overall survival (OS) in AML according to the TCGA data (*P* = 0.006), which was validated by other four independent cohorts. DNA methylation levels of ALOX5AP were significantly lower in AML patients compared to normal samples (*P* < 0.05), as confirmed in the Diseasemeth database and the independent cohort GSE63409. ALOX5AP level was positively associated with genes with proleukemic effects such as PAX2, HOX family, SOX11, H19, and microRNAs that act as oncogenes in leukemia, such as miR125b, miR-93, miR-494, miR-193b, while anti-leukemia-related genes and tumor suppressor microRNAs such as miR-582, miR-9 family and miR-205 were negatively correlated.

**Conclusion:**

ALOX5AP overexpression, associated with its hypomethylation, predicts poorer prognosis in AML.

**Supplementary Information:**

The online version contains supplementary material available at 10.1007/s12672-023-00826-9.

## Introduction

Acute myeloid leukemia (AML) is a group of hematologic malignancies characterized by heterogeneity of biological features and clinical outcomes that can lead to blocked differentiation and uninhibited growth of leukemic primitive cells, thereby limiting the growth of normal blood cells [1]. Recurrence remains a common occurrence in the treatment of AML in spite of current intensive chemotherapy, hematopoietic stem cell transplantation and growing targeting agents [2, 3]. The identification of new reliable molecular markers is still an unsatisfied clinical need for accurate individualized treatment in AML.

Leukotrienes are derived from the nuclear membrane and are key immunomodulatory and pro-inflammatory lipid mediators [4], produced and excreted in response to various immune stimuli that have been implicated in several aspects of carcinogenesis: promoting chemotaxis and the activation of leukocytes [5]. The leukotriene pathway begins with the oxidation of arachidonic acid to leukotriene A4 (LTA4) via lipoxygenase-activating protein (ALOX5AP), which can then be converted to leukotriene B4 (LTB4) via leukotriene A4 hydrolase or coupled to reduced glutathione via leukotriene C4 [6]. Although ALOX5AP has been reported to possess important prognostic significance in several cancer types, including serous ovarian cancer [4], thyroid cancer [7], lung adenocarcinoma [8], and glioma [9]. In recent years, only one study has reported that ALOX5AP gene locus polymorphism may be associated with susceptibility to acute myeloid leukemia [10] via polymerase chain reaction-restriction fragment length polymorphism (PCR–RFLP) combined with directly sequencing, and there are currently no reports on the correlation between expression and methylation levels. There has also been no research indicating the role of ALOX5AP in AML. In the present study, we use bioinformatics analysis, Real-time quantitative PCR (RQ-PCR), and Targeted bisulfite sequencing to analyzed the alteration of ALOX5AP and explored its clinical impact in AML.

## Materials and methods

### Patients and samples

This study was approved by the Ethics Committee and Institutional Review Committee of Jiangsu University Affiliated People's Hospital. Among the 228 patients studied, Real-time quantitative PCR (RQ-PCR) was used to obtain ALOX5AP expression data from 102 patients, including 82 de novo AML patients and 20 healthy donors. ALOX5AP methylation levels were detected in 128 patients, including 102 de novo AML patients and 26 healthy donors through Targeted bisulfite sequencing. BM was collected after the participants signed the informed consents. All patients were diagnosed and classified according to the French-American-British (FAB) classification and the World Health Organization (WHO) criteria. The treatment protocol was described as previously [11].

### Data mining on the TCGA and GEO databases

This study obtained the expression of ALOX5AP in tumor and normal tissues through The Cancer Genome Atlas (TCGA, https://cancergenome.nih.gov/) and Tumor Immune Estimation Resource (TIMER, https://cistrome.shinyapps.io/timer/) databases. The Gene Expression Omnibus (GEO, http://www.ncbi.nlm.nih.gov/geo/) database GSE24006 and GSE63270 was utilized to confirm differential expression of ALOX5AP between AML patients and normal individuals (see Additional file 1: Table S1 for details). Comparing the methylation levels of ALOX5AP in normal controls and AML patients through Diseasemeth database and GSE63409. Furthermore, we conduct an investigation into the impact of ALOX5AP on the prognosis of AML patients through GSE10358, GSE37642, GSE106291, and GSE146173 databases. To determine ALOX5AP mRNA levels in AML, we utilized the R packages edgeR and ggplot2 to normalize, label, and compare raw count data with those of normal controls. While the package "limma" was used for gene expression and DNA methylation microarray data, in addition to the Pheatmap (1.0.12) package for hierarchical clustering analysis and visualization, and the Maftools (1.6.15) [12] mutation analysis package. Table 1 presents additional characteristics of the TCGA participants included in this analysis.

**Table 1:** Patient characteristics with respect to ALOX5AP expression

Patient characteristics	AML (TCGA dataset)		
	High *ALOX5AP* (n = 91)	Low *ALOX5AP* (n = 92)	*P*
Age, years			0.002
Median	61	52.5	
Range	18–88	21–82	
Sex (male/female)	50/41	48/44	0.707
WBC count, × 10^9^/L			0.005
Median	22.9	11.5	
Range	0.8–298.4	0.5–223.8	
BM blasts, %			0.471
Median	73	72	
Range	30–99	32–100	
PB blasts, %			0.022
Median	22	42.5	
Range	0–98	0–97	
Karyotype			0.030
Favorable	10	24	
Intermediate	61	44	
Adverse	18	22	
Unknown	2	2	
FLT3-ITD*			0.065
Present	9	18	
Absent	82	74	
NPM1*			0.085
Mutated	29	19	
Wild-type	62	73	
*CEBPA*			0.370
Single mutated	3	4	
Double mutated	4	1	
Wild-type	84	87	
IDH1*			0.005
Mutated	3	14	
Wild-type	88	78	
IDH2*			0.603
Mutated	10	8	
Wild-type	81	84	
RUNX1*			0.771
Mutated	8	7	
Wild-type	83	85	
DNMT3A*			0.463
Mutated	24	20	
Wild-type	67	72	
TP53*			0.982
Mutated	8	8	
Wild-type	83	84	

AML: acute myeloid leukemia; TCGA: The Cancer Genome Atlas; WBC: white blood cells; BM: bone marrow; PB: peripheral blood; ITD: internal tandem duplication

^*^The median expression value was used as a cut point

### RNA isolation, reverse transcription, and RQ-PCR

Total RNA was extracted by using TRIzol reagent (Invitrogen, Carlsbad, CA, USA). The extraction and reverse transcription have been described previously [13]. RQ-PCR was performed to detect ALOX5AP expression by using a 7500 Thermal cycler (Applied Biosystems, CA). The procedure of RQ-PCR was conducted as described previously [14, 15]. PCR primers were designed using Primer Premier 6 (Premier Biosoft, Palo Alto, CA, USA), and the primer sequences were listed in Additional file 1: Table S2. The relative expression levels of ALOX5AP were calculated by 2-ΔΔCT method and normalized to the ABL housekeeping gene.

### Targeted bisulfite sequencing

Genomic DNA was extracted from bone marrow cells with Puregene Blood Core Kit B (QIAGEN Sciences, MD, USA), then DNA was subjected to bisulfite modification by EZ DNA Methylation-Gold Kit (ZYMO, CA, USA), following the manufacturer’s instructions. Primers were designed using primer 3 (http://primer3.ut.ee/) based on the bisulfite converted DNA. Multiplexed PCR amplification of the target sequence, followed by index PCR, and the PCR products were further purified to generate sequencing library. Paired-end sequencing (2×150 bp) was performed on the Illumina MiSeq platform (Illumina, San Diego, CA, USA). After quality assessment and filtering, paired reads generated from the Illumina MiSeq platform were merged using FLASH (Fast length adjustment of short reads) [16]. The merged reads were then aligned (mapped) to the human GRCh37/ hg19 genome assembly (in silico bisulfite-converted human reference genome [GRCh37]) using blast+ [17]. CpG methylation values were calculated as methylated reads counts/total read counts of every CG point.

### Gene Ontology and KEGG enrichment analysis

Based on the TCGA database, 1149 differentially expressed genes and 75 miRNAs associated with ALOX5AP expression were identified by comparing the transcriptome of the ALOX5AP low expression group and the high expression group. Through GO and KEGG enrichment analysis, it was demonstrated that these genes participate in various biological processes.

### Immunocell infiltration analysis

To explore the potential relationship between ALOX5AP expression and immune cell infiltration, we explored the CIBERSORT algorithm to analyze the TCGA cohort. Meanwhile, we used estimation algorithms to calculate the immune scores of all AML data in the TCGA database and demonstrated their correlation with ALOX5AP expression.

### Statistical analysis

We conducted statistical analysis using several software programs, including R version 4.0 (https://www.r-project.org), SPSS 22.0 software package (IBM Corp, Armonk, NY, USA) and GraphPad Prism 8.0. Based on the median level of ALOX5AP expression, we divided the patients into high and low expression groups. Survival probabilities of the two groups were estimated by the Kaplan–Meier method. To test for the significance of the differences across groups, Chi square tests or Fisher’s exact test was used for categorical data. For continuous data, a nonparametric test such as the Mann–Whitney U-test was used. Spearman correlation analysis was performed to determine the association between two continuous variables. A *p-*value of less than 0.05 was considered statistically significant.

## Results

### ALOX5AP expression in AML

**Fig. 1:**
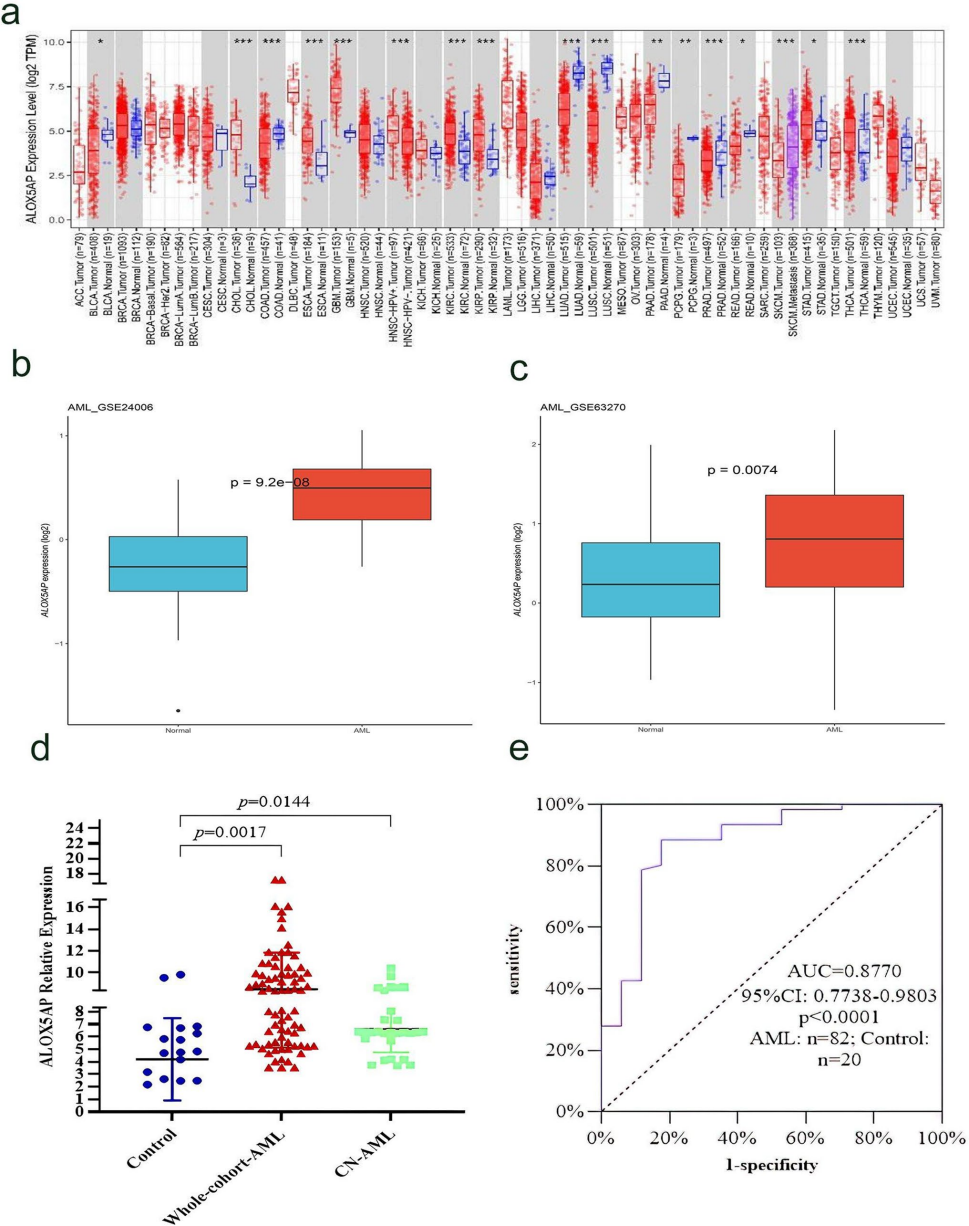
A study of ALOX5AP expression levels in AML and other types of cancers among the TCGA and GEO dataset. **a** The differential expression between the tumor and adjacent normal tissues for ALOX5AP across all TCGA tumors via TIMER. **b**, **c** The expression changes of ALOX5AP in AML and normal bone marrow cells from GSE24006 (n = 31 normal and 20 AML) and GSE63270 (n = 42 normal and 62 AML), **p < 0.01. **c** Relative expression levels of ALOX5AP in AML patients and controls. The transcript levels of ALOX5AP in controls, whole-AML patients, and CN-AML patients were evaluated by RQ-PCR. Horizontal lines represent the median level of ALOX5AP expression in each group. n = 102; one-way ANOVA. The data are expressed as the mean ± SEM; *P* < 0.05 among all groups.** d** Roc curve analysis of ALOX5AP for distinguishing AML patients from controls. n = 102; Mann–Whitney u-test, ***p < 0.001

**Fig. 2:**
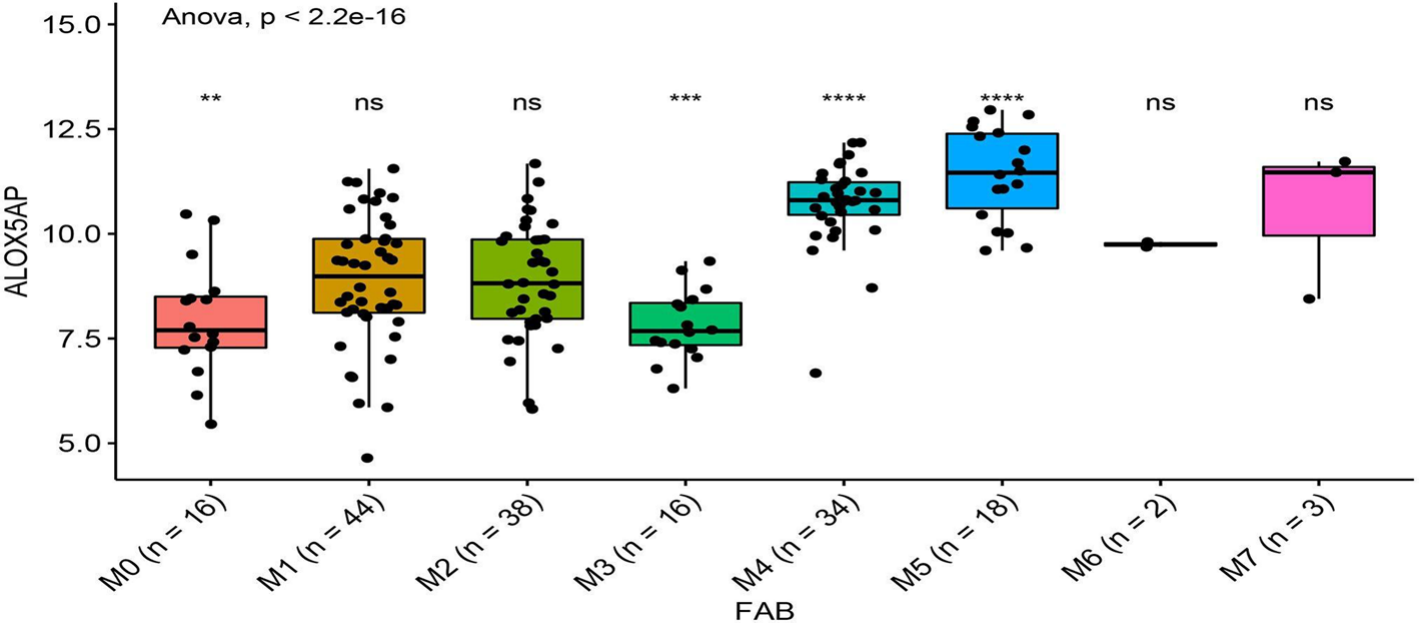
Expression patterns of ALOX5AP in different FAB AML subtypes from the TCGA cohort. n = 171, ns: not significant, *p < 0.05, **p < 0.01, ***p < 0.001

We examined the expression of ALOX5AP in TCGA using the TIMER database. ALOX5AP expression was higher in tumor tissues than in normal tissues from glioblastoma multiforme (GBM), lymphoid neoplasm diffuse large B-cell lymphoma (DLBC) and AML (Fig. 1a). Further analysis on GSE24006 and GSE63270 datasets has confirmed the results (*P* < 0.001 and *P* < 0.001, respectively, Fig. 1b, c). For further verification, the results of RQ-PCR reveal that ALOX5AP expression was significantly increased in AML patients (3.55–17.16, median 8.404) compared with controls (1.21–9.82, median 2.627) (*P* = 0.0017, Fig. 1d). Moreover, the increased expression of ALOX5AP was also found in cytogenetically normal acute myeloid leukemia (CN-AML) patients (*P* = 0.0144, Fig. 1d). ROC curve analysis revealed that the AUC value was 0.8770 (95% CI 0.7738–0.9803, *P* < 0.0001) in our cohort (Fig. 1e), which suggested the ALOX5AP expression level might be a potential biomarker to discriminate AML from controls. Moreover, ALOX5AP expression was significantly higher in monocytic leukemias (M4 and M5 subtypes according to FAB classification, Fig. 2).

### Differences in clinical characteristics between the low and high ALOX5AP expression groups

**Fig. 3:**
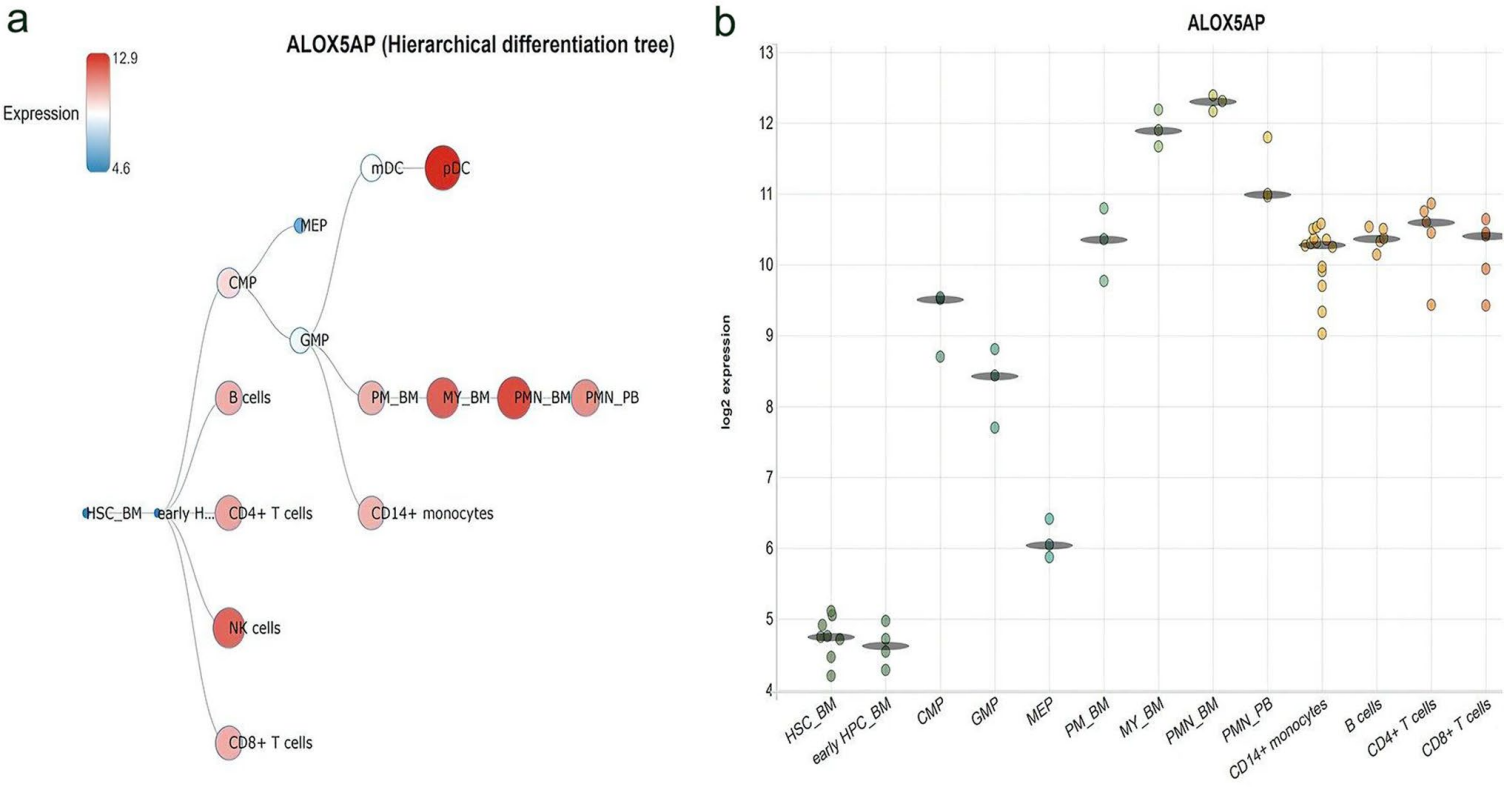
The expression of ALOX5AP in cell types. **a** Hierarchical difference tree about cell types with ALOX5AP expression. **b** The expression of ALOX5AP in a microarray in cell types. Horizontal lines represent the median expression for each class of cells

We investigated the clinical characteristics of ALOX5AP in AML patients from public TCGA database. Based on the median level of ALOX5AP transcript, we divided the AML patients into high and low expression groups (Table 1). No significant differences were observed in sex and BM blast percentage (*P* > 0.05). However, patients with high ALOX5AP expression were more likely to be older (*P* = 0.002), with higher white blood cell (WBC) counts (*P* = 0.005). We also found that patients with low expression of ALOX5AP carried more favorable karyotype (*P* = 0.03). With respect to known prognostic markers, patients with low ALOX5AP expression more often had IDH1 mutations (*P* < 0.05). In order to explore the changes of ALOX5AP expression patterns in the process of hematopoiesis, we analyzed the BloodSpot and HemaExplorer databases, we found that ALOX5AP transcripts was low in hematopoietic stem cells from bone marrow (BM HSCs), with a sharp growth in committed progenitors including common myeloid progenitor (CMP) and granulo-monocyte progenitor (GMP), then remained at a high and stable level during myeloid maturation (Fig. 3).

### Comparison of ALOX5AP methylation levels in AML and normal samples

**Fig. 4:**
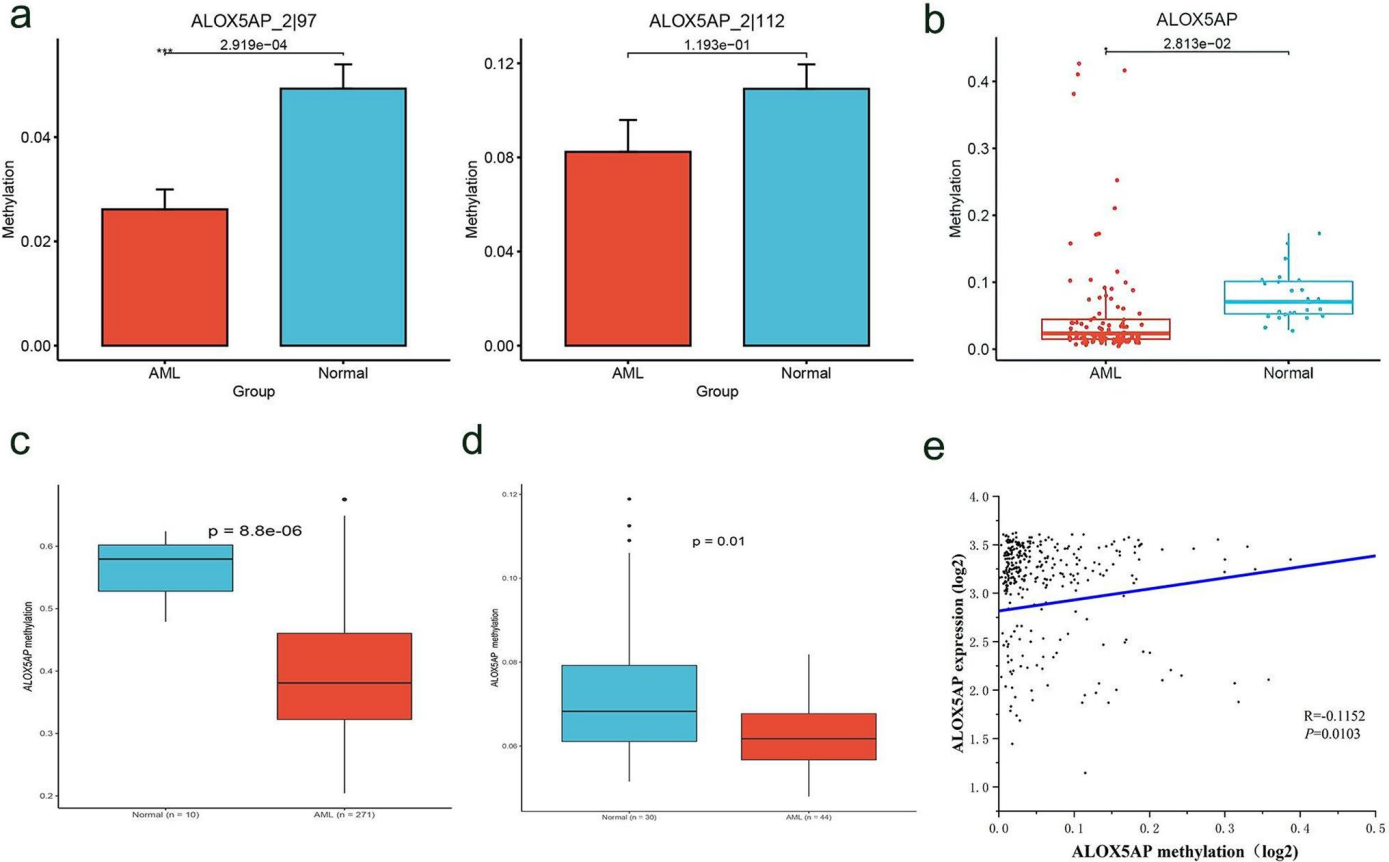
Methylation patterns of ALOX5AP in AML. **a**, **b** ALOX5AP methylation levels are determined by targeted bisulfite sequencing. Detailed methylation data of methylation levels at 2 CpG sites in the ALOX5AP first exon (chr13: 30735647–30735662) are provided in Additional file 1. n = 26 normal and 102 AML; Mann–Whitney u-test. The data are expressed as the mean ± SEM; *p < 0.05, **p < 0.01. **c, d** ALOX5AP methylation levels in normal and AML patients for the Diseasemeth database (n = 10 normal and 271 AML) and GSE63409 (n = 30 normal and 44 AML), **p < 0.01. **e** Scatterplot showing the correlation between ALOX5AP methylation and ALOX5AP expression in AML patients. R = − 0.1152, *p < 0.05

To assess the methylation levels of ALOX5AP in primary AML samples, we used Targeted bisulfite sequencing to measure the methylation levels of 2 CpG sites in the first exon of ALOX5AP (chr13: 30735647–30735662) (*P* < 0.01, Fig. 4a). Detailed methylation data for this sequenced region was provided in Additional file 2. The β values of the 2 CpG sites were then averaged as the DNA methylation level of ALOX5AP gene, which was significantly lower in AML patients compared with healthy controls (*P* < 0.001, Fig. 4b). Furthermore, the methylation results were also confirmed in the Diseasemeth database *(P* = 8.8e−06, Fig. 4c) and the cohort GSE63409 *(P* = 0.01, Fig. 4d). In addition, ALOX5AP methylation levels were negatively correlated with mRNA levels (R = -0.1152, *P* = 0.0103, Fig. 4e).

### The impact of ALOX5AP expression on prognosis in AML patients

**Fig. 5:**
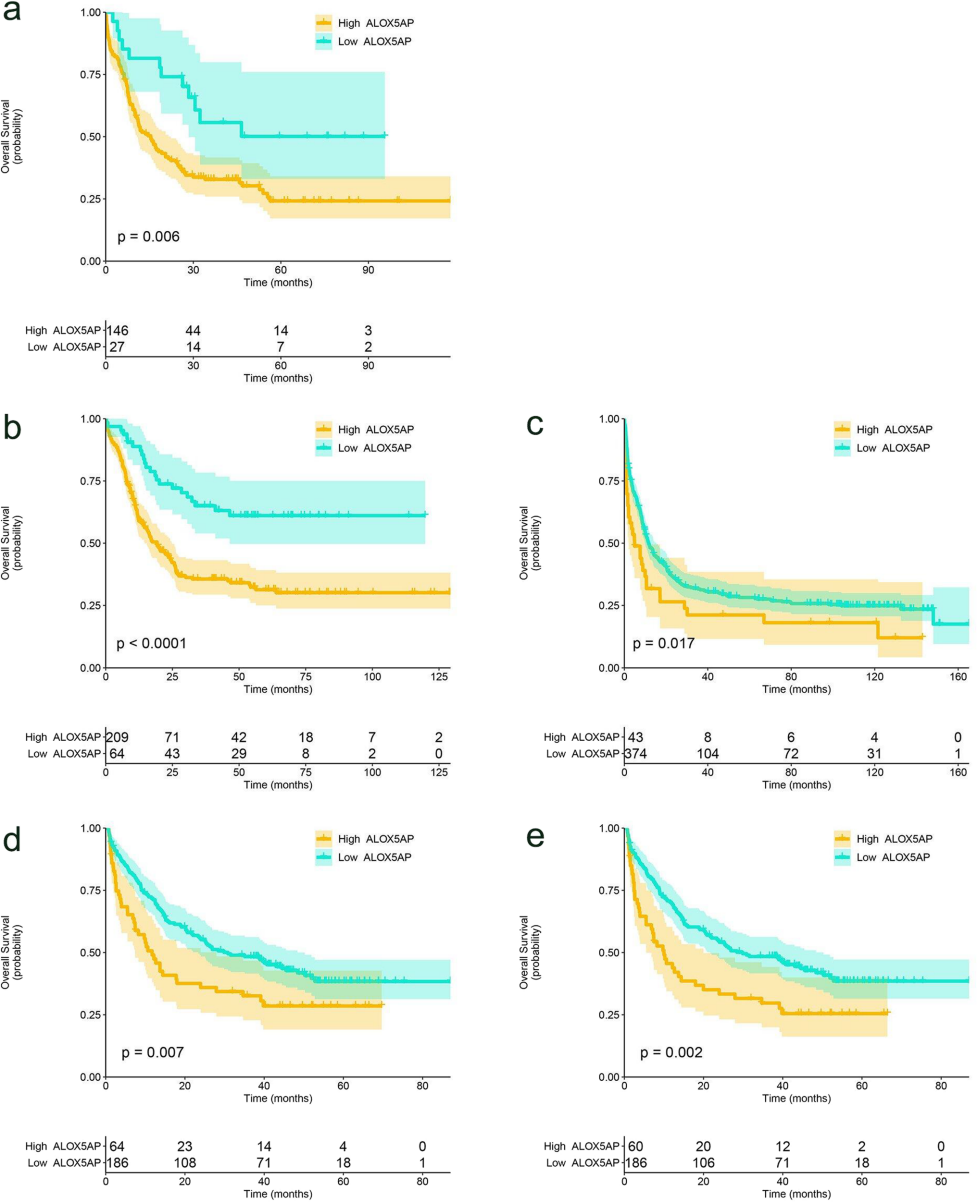
The impact of ALOX5AP expression on overall survival in AML patients. **a** Kaplan–Meier survival curves of OS in AML patients from TCGA cohort (n = 173). **b–e** Kaplan–Meier survival curves of OS in AML patients from GSE10358 (n = 273), GSE37642 (n = 417), GSE106291 (n = 250), and GSE146173 (n = 246) cohort. *p < 0.05, ** *p* < 0.01, ***p < 0.001

The overall survival of patients with high ALOX5AP expression in the TCGA AML cohort was significantly worse (*P* = 0.006, Fig. 5a). Furthermore, we evaluated four independent datasets (GSE10358, GSE37642, GSE106291 and GSE146173), all of which indicated that elevated ALOX5AP mRNA levels were significantly associated with poor prognosis in AML patients (Fig. 5b–e).

### Distinct gene—and microRNA-expression signatures associated with ALOX5AP expression in AML

**Fig. 6:**
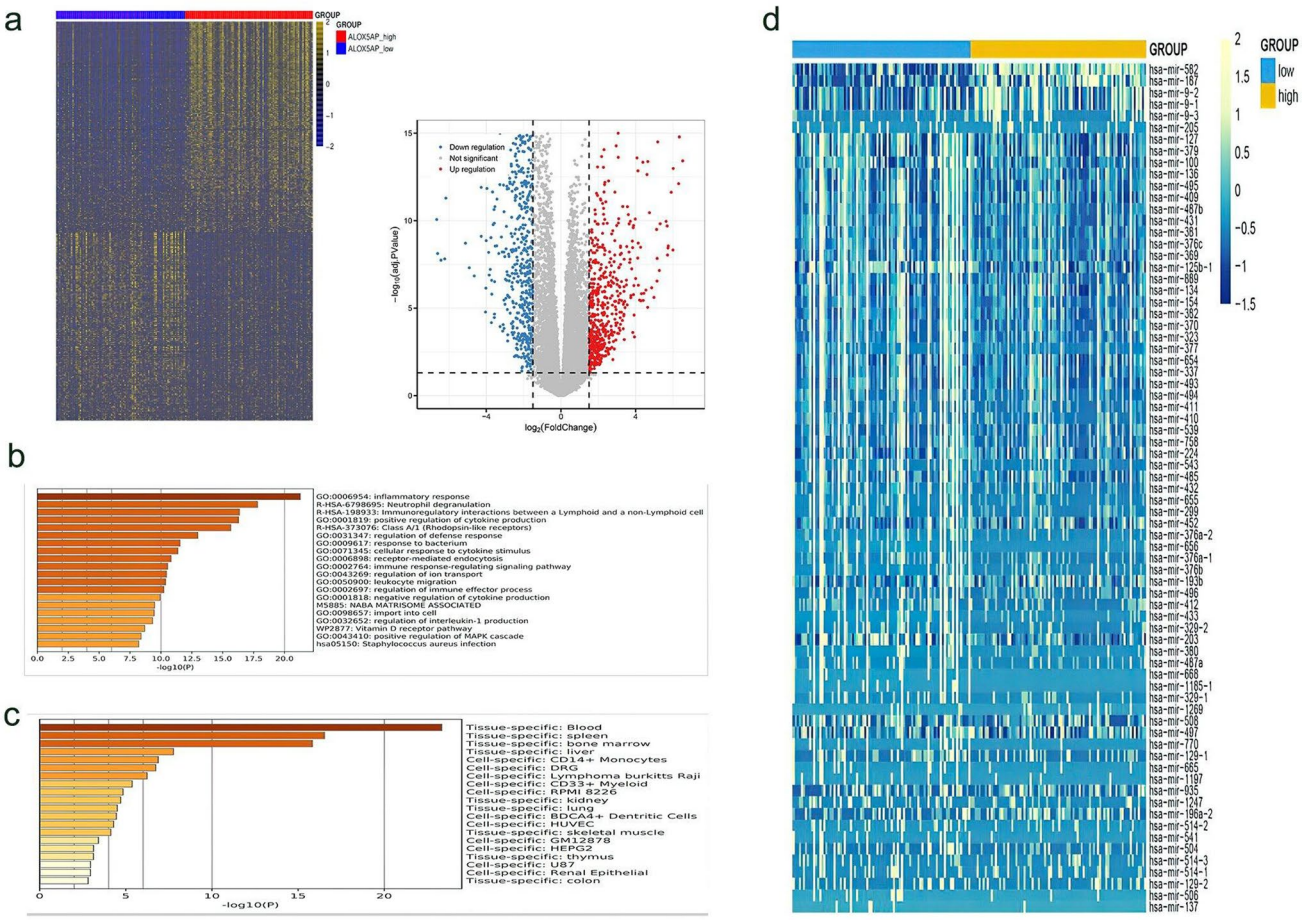
Gene/microRNA signatures associated with ALOX5AP expression. **a** Left: heatmap showing the gene expression signature associated with ALOX5AP expression. Right: volcano plot showing gene expression differences between patients with low and high ALOX5AP expression. (FDR < 0.05, |log2 FC|> 1.5). **b** Analysis of GO and KEGG pathway associated with ALOX5AP expression. **c** Enrichment of differentially expressed genes in tissues and cells. **d** Heatmap showing the microRNA expression signature associated with ALOX5AP expression (FDR < 0.05, |log2 FC|> 1.5). Up regulated and down regulated microRNAs mentioned in the text are indicated

The transcriptomes were compared between ALOX5AP low and high expression groups based on the TCGA database. A total of 1149 differential genes were identified (FDR < 0.05, | log2 FC|> 1.5; Fig. 6a; Additional file 3), with 544 genes positively and 605 negatively associated with the ALOX5AP expression levels, using the median as the cut-off value. To extend the observation that clinical features differ between AML samples with low versus high ALOX5AP expression, we first compared the transcriptomes of low ALOX5AP expressed group with those of high expression. Among these positively related genes, a number of oncogenes comprise the main ones. These contain known cancer-testis antigen genes (MEGA5, MEGA1, TEX15, PNMA5 and ROR1) and a number of new candidate genes (PAX2, NTN4, NTNG2 and PDE10A), these genes can be used as tumour markers or potential therapeutic targets with some clinical value. In parallel, we identified a number of epithelial-to-mesenchymal transition (EMT) inducers, including MMP2, TWIST, HIF and PROX1, PROX1 [18] and MMP2 [19] are thought to be targets of the Wnt signalling pathway. Besides, MMPs could be involved in cell displacement and vascular leakiness, recent studies have suggested MMP inhibition could be a promising complementary therapy to reduce AML growth and limit HSPC loss and BM vascular damage caused by MLL-AF9 and possibly other AML subtypes [20]. Oncogenes were significantly enriched in genes co-expressed with ALOX5AP. In contrast, a number of reported tumour suppressors were found in genes negatively associated with ALOX5AP expression, including negative regulators of EMT, such as ARHGAP29, RBM47 and KLF4; There's also the Wnt antagonist gene DKK2 in the mix, it has been proposed that overexpression of KIF4 may inhibit EMT in gastric cancer cells through the Wnt/β-catenin signalling pathway[21].

Furthermore, Gene Ontology and KEGG enrichment analysis showed that these genes are involved in several biological processes (Fig. 6b). The most enriched were inflammatory responses, neutrophil degranulation, and immunoregulatory interactions between a Lymphoid and a non-Lymphoid cell, which confirmed that ALOX5AP is required for the synthesis of leukotrienes and is involved in various types of inflammatory responses. Several of these GO entries occurred in biological processes, such as positive regulation of cytokine production and positive regulation of MAPK cascade, all these implied that ALOX5AP-related genes are pro-carcinogenic. In addition, these DEGs were significantly enriched in blood and bone marrow (Fig. 6c), which further suggested a potential role for ALOX5AP in haematological tumours.

We also obtained ALOX5AP-associated microRNA expression profiles containing 75 microRNAs (FDR < 0.05, |log2 FC |> 1.5; Fig. 6d; Additional file 4). Among the 75 miRNAs, 68 miRNAs were up-regulated and 7 were down-regulated in ALOX5AP^high^ patients. Among the down-regulated microRNAs, miR-582, miR-187, miR-9–2, miR-9–1 and miR-9–3, which could inhibit the development of a variety of tumours, including bladder, gastric and lung cancers [22–24]. We also found that miR-582 and miR-187 were involved in inhibiting the EMT process [25, 26]. Most up-regulated miRNAs acted as oncogenes in tumour diseases, while in haematological tumours, miR125b, miR-93 and miR-196 could promote leukemogenesis through increasing the leukemic stem/progenitor cell population, promoting cell proliferation, blocking cell differentiation, and diminishing cell apoptosis [27].

### Correlation between ALOX5AP and immunocytes infiltration

**Fig. 7:**
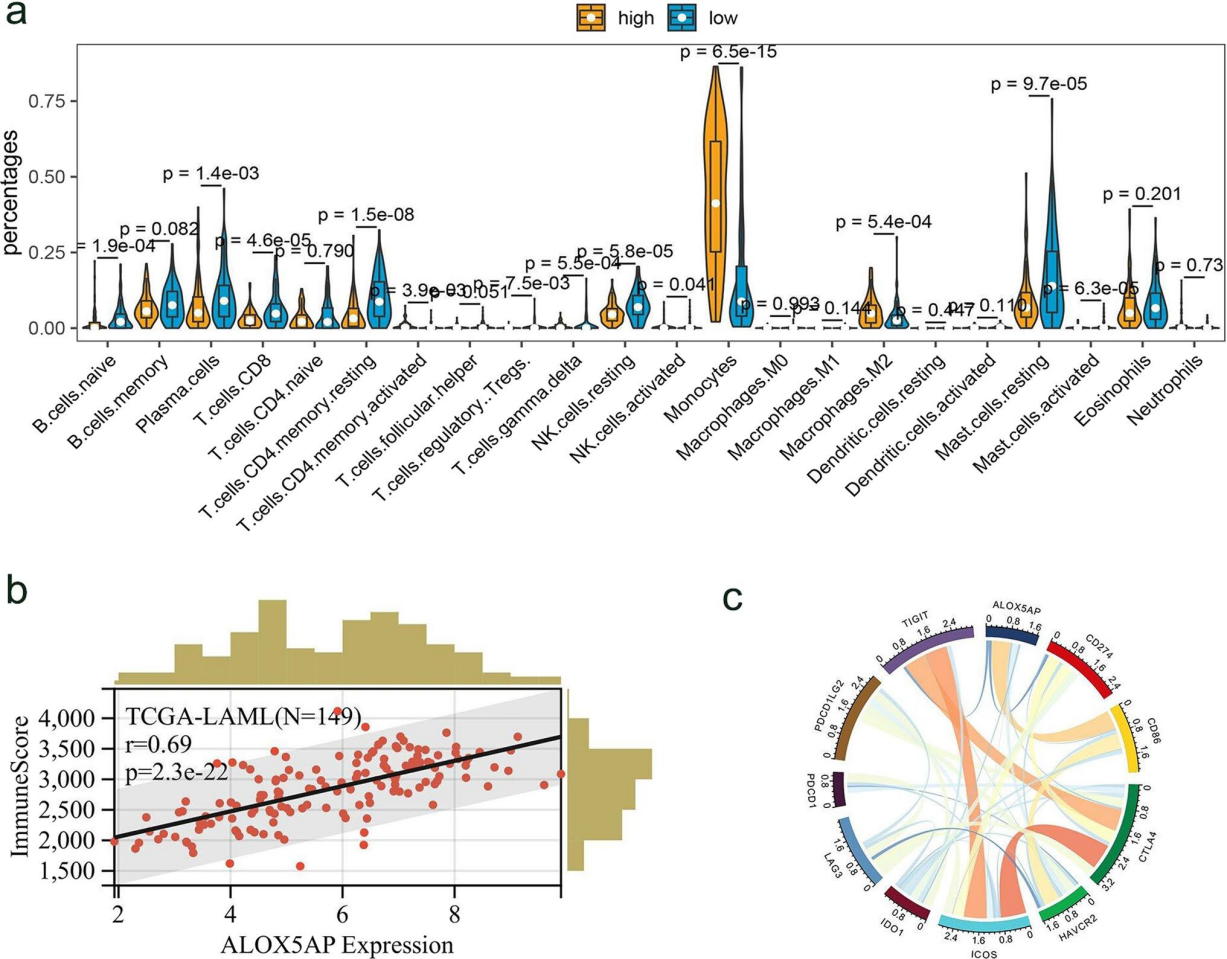
Tumor-infiltrating immune cells in the AML microenvironment and checkpoint genes associated with ALOX5AP expression. **a** The relation between ALOX5AP expression and immune cell infiltration. Violin plot showing the differences of immune cell fractions between patients with low and high ALOX5AP expression. The overall immune cell compositions were estimated by CIBERSORT across TCGA microarray. **b** Correlation of ALOX5AP expression and immune score in AML across TCGA database. **c** Correlation betweenALOX5AP and other immune checkpoints in AML across TCGA database

Tumor tissue is composed of various types of cells, including stromal cells, fibroblasts, and immune cells. These cells constitute the tumor’s microenvironment. Therefore, we focused on the correlation between ALOX5AP expression and the level of immune cell infiltration. We evaluated the relationship between ALOX5AP expression and tumor-infiltrating immune cells in the AML microenvironment in the TCGA database using the CIBERSORT algorithm. ALOX5AP expression was significantly positively correlated with monocytes and macrophages M2 (*P* < 0.001, Fig. 7a). This is consistent with the significantly elevated expression of ALOX5AP in monocytic leukaemia (M4 and M5 subtypes of the FAB classification) mentioned above. In contrary, those cells performing anti-tumor responsiveness (i.e., activated CD4 T cells, effector memory CD4 T cells, and CD8 T cells) were significantly negatively correlated ALOX5AP expression (*P* < 0.001). Meanwhile, we found that ALOX5AP significantly enhanced the immune score in AML (*P* < 0.05) (Fig. 7b). All these data suggested that ALOX5AP was closely related to tumor immune infiltration. Furthermore, given that immune checkpoints are promising therapeutic targets for cancer therapy, we also evaluated the relationship between ALOX5AP and the set of checkpoint genes (Fig. 7c). We found that the more widely studied immune checkpoint CD86 was significantly positively associated with ALOX5AP expression, While HAVCR2 (TIM-3), CD274 (PD-L1), and TIGIT were negatively correlated with ALOX5AP expression.

## Discussion

Many studies have identified various genes and molecules that can serve as biomarkers for the diagnosis and treatment of AML, including: several FLT3 inhibitors in the clinic [28], menin inhibitor, preliminary results in relapsed/refractory NPM1 mutant and KMT2Ar AML have shown on-target effects [29], venetoclax combined with FLT3- and MCL-1 inhibitors, to enhance venetoclax-induced death of leukemia cells [30], furthermore, T cells and natural killer (NK) cells engineered with chimeric antigen receptors (CARs) currently being tested in preclinical and clinical settings targeting AML-associated antigens [31]. Although now with an improved understanding of pathophysiology, improvements in measurement technology and at least 10 recently approved therapies have led to revamping the diagnostic, prognostic, and therapeutic landscape of AML [32], the optimal sequencing of therapy, the combination of therapies, and whether maintenance therapy is effective in patients who achieve remission and are unable to receive allogeneic HSCT remain urgent questions [33].

Our study was conducted by searching TCGA and GEO databases, which showed that ALOX5AP was significantly upregulated in AML patients compared to a normal controls, and that high ALOX5AP expression associated with DNA hypomethylation were correlated with poor prognostic clinical and biological features. Our study further confirmed that ALOX5AP was up-regulated in whole-AML as well as CN-AML compared with normal controls. Among FAB subtypes, ALOX5AP expression in M4 and M5 was significantly higher than that in other subtypes, acute monoblastic/monocytic leukemia (AMoL), previously defined as M5 according to FAB classification, is one of the most common subtypes of AML in children, representing ~ 15–24% of all pediatric AMLs, new diagnostic markers need to be developed to obtain a clear distinction between leukemia and reactive monocytes [34]. During hematopoiesis, the expression of ALOX5AP was low in BM_HSCs, then increased rapidly and was at a range of high level fluctuations in the following process, it reflects that ALOX5AP is likely to play a role in the specific stage of differentiation. With respect to the prognostic relevance, we found high ALOX5AP expression predicted inferior OS in the whole TCGA cohort. The predictive value for OS was further validated in four independent cohorts. In addition, ALOX5AP DNA methylation also affected gene expression levels, as we showed by targeted bisulfite sequencing that ALOX5AP gene methylation levels were significantly lower in AML patients compared to healthy controls, this was also validated by comparison of the Diseasemeth database and independent cohorts of GSE63409 normal and AML patients with ALOX5AP methylation levels. DNA methylation is one of the most important epigenetic mechanisms for regulating gene expression, which is highly dynamic during development and maintains specificity in somatic cells. Abnormal DNA methylation patterns are closely related to human diseases, including cancer [35]. In general, hypermethylation of specific gene promoter often leads to gene silencing. On the contrary, gene promoter hypomethylation often goes along with gene activation due to more open chromatin structure [36]. We can preliminarily infer that ALOX5AP expression may be regulated by its methylation through the scatter plot of the correlation between ALOX5AP methylation and ALOX5AP expression.

We derived ALOX5AP-associated gene/microRNA expression signatures in AML. One important feature was the identification of several EMT-related genes, including MMP2, TWIST, HIF and PROX1. Notably, TWIST reported as a novel poor prognostic factor in acute myeloid leukemia [37], HIF accelerates disease progression in mouse models of leukaemia and lymphoma, it has not been confirmed whether it is a poor prognostic factor for human AML [38]. It was somewhat surprising, however, modulators of EMT have recently emerged as novel players in the field of leukemia biology. For example, ectopic expression of Snail in haematopoietic cells was found to predispose mice to acute myeloid leukaemia by interacting with the histone demethylase KDM1A/LSD1 [39], this is particularly important given the current interest in the use of LSD1 inhibitors for the treatment of many different malignancies, including AML. Furthermore, ZEB2 is a classical EMT regulator that has been shown to play an important role in haematopoiesis and leukaemia transformation [40], Wang et al. demonstrated the oncogenic role of ZEB1/2 in AML by showing that inactivation of ZEB2 alone or ZEB1/2 together improves survival in the secondary MLL-AF9 acute myeloid leukaemia (AML) model [41], indicating the cellular environment dependence of EMT regulators. Another interesting finding was that several DEGs positively associated with ALOX5AP expression were identified including EPHA5, PDPN, PAX2 and RUNX1T1 promote tumor metastasis and invasion through activation of the Wnt signaling pathway, which to our knowledge is required for leukemic stem cell development in AML [42], it has been shown that the Wnt/β-linked protein signaling pathway is involved in the growth and proliferation of CML cells and is closely related to the self-renewal ability of CML leukemic stem cells [43], while the Wnt signaling pathway also plays a role in multidrug resistance in hematologic malignancies [44]. In contrast, the Wnt antagonist DKK2 was found in genes negatively associated with ALOX5AP expression, our earlier study also identified the prognostic significance of some other Wnt antagonists in AML [45], which highlighted the importance of dysregulated Wnt signalling in leukemogenesis. It was therefore reasonable to speculate that overexpression of ALOX5AP may contribute to the activation of the Wnt pathway and enhancement of leukaemia cell proliferation.

MicroRNAs positively associated with ALOX5AP expressions, such as miR125b, miR-93, miR-494, and miR-193b, acted as oncogenes in leukemia. In T-ALL, circ_0000745 can promote T-ALL cell proliferation and inhibit apoptosis by targeting miR-193b to upregulate NOTCH1 [46]. Notably, miR-495 was identified as a predicted miRNA that could directly target ALOX5AP [47]. Among the down-regulated microRNAs, lncRNA-MALAT1 inhibited ALL cell proliferation and promoted apoptosis by targeting the miR-205-PTK7 pathway, providing a potential therapeutic target for ALL [48]. Further evidence of the pro-cancer role and negative prognostic significance of ALOX5AP in haematological diseases.

The tumor microenvironment in AML is characterized by immunosuppression, which promotes immune tolerance and the immune escape of malignant cells [49]. The main components of the AML bone marrow microenvironment (BMM) include T cells, B cells, and NK cells [50]. In AML, the immunoregulatory network in the BMM is an important contributor to cancer progression [51]. We speculated that immune cell infiltration has a significant impact on the immune microenvironment, tumor development, treatment strategies, and likelihood of recurrence. Therefore, studying the infiltration of immune cells in tumors may provide valuable insights into the efficacy and mechanism of immunotherapy for AML. Our study evaluated the relationship between ALOX5AP expression and 22 immune cell infiltration. ALOX5AP expression and Monocytes, Macrophages M2 were significantly positively correlated. Combined with the previous mention, the expression of ALOX5AP is significantly increased in monocytic leukemia (M4 and M5 subtypes according to FAB classification), does ALOX5AP play a more specific and significant role in monocytic leukemia, which needs further confirmation. In addition, upregulation of immune checkpoint by infiltrating immune cells is also a key factor in cancer progression. Studies have found that CD86 is overexpressed in many cancers, especially in AML, and its high expression in AML cell lines has been shown to be associated with poor prognosis [52]. In summary, it further demonstrates the important role of ALOX5AP in AML.

Nevertheless, there were some limitations to our study. Firstly, most of our samples were obtained from public databases, and the validation sample size was relatively small, requiring a large number of patient samples for subsequent validation. Secondly, further validation is needed through in vivo and in vitro studies.

## Conclusion

In summary, our study provides evidence that ALOX5AP expression is high in AML, and that high expression is associated with poor prognosis in AML. Moreover, ALOX5AP methylation level in AML is significantly decreased. We hope that the unique genetic pattern of ALOX5AP will contribute to our further understanding of the heterogeneous mechanisms of AML.

### Supplementary Information

Below is the link to the electronic supplementary material

Supplementary file 1 (DOCX 14 KB)

Supplementary file 2 (XLSX 14 KB)

Supplementary file 3 (ZIP 1430 KB)

Supplementary file 4 (ZIP 16 KB)

## Data Availability

The datasets analyzed in this study are available in the following open access repositories: TCGA, http://www.cbioportal.org, http://gdac.broadinstitute.org. GEO, https://www.ncbi.nlm.nih.gov/geo/ (GEO accession numbers: GSE24006, GSE10358, GSE37642, GSE106291, GSE146173, GSE63270, and GSE63409). BloodSpot, http://servers.binf.ku.dk/bloodspot/. The in-house methylation data was provided as Additional file 2. Other datasets are available from the corresponding author upon reasonable request.

## Abbreviations

BM: Bone marrow
AML: Acute myeloid leukemia
CN-AML: Cytogenetically normal acute myeloid leukemia
ROC: Receiver operating characteristic
FAB: French-American-British
WHO: World Health Organization
OS: Overall survival
TCGA: The Cancer Genome Atlas
GEO: Gene Expression Omnibus
TIMER: Tumor Immune Estimation Resource
ALOX5AP: Lipoxygenase-activating protein
RT-qPCR: Real-time quantitative PCR
LTA4: Leukotriene A4
LTB4: Leukotriene B4
GBM: Glioblastoma multiforme
CMP: Common myeloid progenitor
GMP: Granulo-monocyte progenitor
DLBC: Lymphoid neoplasm diffuse large B-cell lymphoma
BM HSCs: Hematopoietic stem cells from bone marrow
AMoL: Acute monoblastic/monocytic leukemia
EMT: Epithelial-to-mesenchymal transition
BMM: Bone marrow microenvironment
