# Supplementary material for: ALOX5AP is a new prognostic indicator in acute myeloid leukemia
Source: Discov Oncol. 2023 Nov 23;14:210. doi: 10.1007/s12672-023-00826-9 (PMC10667204; doi:10.1007/s12672-023-00826-9)
Supplement: Supplementary file 1 — (DOCX 14 KB) [file 12672_2023_826_MOESM1_ESM.docx]

| **Table S1.** **Details of public datasets used in this study** | | | | | |
| --- | --- | --- | --- | --- | --- |
| Dataset | Data type | Platform(s) | Sample type | No. of samples | Reference |
| TCGA | RNA-seq | Illumina HiSeq 2000 | AML | 173 | Ley, TJ, et al (2013) |
| TCGA | miRNA-seq | Illumina Genome Analyzer | AML | 188 | Ley, TJ, et al (2013) |
| GSE24006 | microarray | Affymetrix U133 Plus 2.0 | AML, NBM | 54 | Gentles, AJ, et al (2010) |
| GSE10358 | microarray | Affymetrix U133 Plus 2.0 | AML | 304 | Xiang Z, Walgren R, et al (2008) |
| GSE37642 | microarray | Affymetrix U133 Plus 2.0 | AML | 140 | Herold T, Jurinovic V, et al (2018) |
| GSE106291 | microarray | Illumina HiSeq 1500 | AML | 250 | Herold T, Jurinovic V, et al (2018) |
| GSE146173 | microarray | Illumina HiSeq 1500 | AML | 246 | Bamopoulos SA, et al(2020) |
| GSE63270 | microarray | Affymetrix U133 Plus 2.0 | AML, NBM | 104 | Jung, N, et al (2015) |
| GSE63409 | DNA methylation array | Illumina HumanMethylation450 | AML, NBM | 74 | Jung, N, et al (2015) |

**Additional Tables**

| **Table S2.** **Primer sequences used in this study** | | |
| --- | --- | --- |
| Primers | Primer sequence (5’ to 3’) | Predicted product size (bp) |
| RQ-PCR primers | | |
| *ALOX5AP*-forword | CTTCCAGAGGACCGGAACAC | 145 |
| *ALOX5AP*-reverse | CATCAGTCCAGCAAACGCAG |  |
| ABL-forword | TCCTCCAGCTGTTATCTGGAAGA | 118 |
| ABL-reverse | TCCAACGAGCGGCTTCAC |  |
| Targeted bisulfite sequencing primers | | |
| *ALOX5AP*-forword | GGTAGGTTGTGTAGTTGGAGGTAG | 196 |
| *ALOX5AP*-reverse | ACCTAACTTCCAAACAACCATCAA |  |
